# COVID-19-Related Stressors and Clinical Mental Health Symptoms in a Northeast US Sample

**DOI:** 10.3390/ijerph20021367

**Published:** 2023-01-12

**Authors:** Mollie A. Monnig, Samantha E. Clark, Jaqueline C. Avila, Alexander W. Sokolovsky, Hayley Treloar Padovano, Kimberly Goodyear, Elizabeth R. Aston, Carolina L. Haass-Koffler, Jennifer W. Tidey, Jasjit S. Ahluwalia, Peter M. Monti

**Affiliations:** 1 Center for Alcohol and Addiction Studies, Department of Behavioral and Social Sciences, Brown University, Providence, RI 02912, USA; 2 Center for Addiction and Disease Risk Exacerbation, Brown University, Providence, RI 02912, USA; 3 Center for Alcohol and Addiction Studies, Department of Psychiatry and Human Behavior, Brown University, Providence, RI 02912, USA

**Keywords:** COVID-19 pandemic, COVID-19 related stressors, clinical mental health symptoms, psychological distress, anxiety, posttraumatic stress, loneliness, food insecurity

## Abstract

Research has linked specific COVID-19-related stressors to the mental health burden, yet most previous studies have examined only a limited number of stressors and have paid little attention to their clinical significance. This study tested the hypothesis that individuals who reported greater COVID-19-related stressors would be more likely to have elevated levels of anxiety, posttraumatic stress symptoms, and serious psychological distress. Methods: An online survey was administered to a convenience sample from 18 June to 19 July 2020, in US states that were most affected by COVID-19 infections and deaths at the time. Individuals who were 18 or older and residents of five Northeast US states were eligible to participate (N = 1079). In preregistered analyses, we used logistic regression models to test the associations of COVID-19 stressors with symptoms on the Generalized Anxiety Disorder-7 (GAD-7), Impact of Event Scale-Revised, and K6, adjusting for sociodemographic covariates. Results: COVID-19-related stressors (i.e., essential worker status, worry about COVID-19 infection, knowing someone hospitalized by COVID-19, having children under 14 at home, loneliness, barriers to environmental rewards, food insecurity, loss of employment) were associated with meeting thresholds (i.e., positive screening) for anxiety, posttraumatic stress, and/or serious psychological distress. Loneliness and barriers to environmental rewards were associated with all mental health outcomes. Limitations: We used a non-probability sample and cannot assume temporal precedence of stressors with regard to development of mental health symptoms. Conclusions: These findings link specific stressors to the mental health burden of the COVID-19 pandemic.

## 1. Introduction

Increases in mental health problems are among the most pressing consequences brought about by the ongoing COVID-19 pandemic. The pandemic may have deleterious effects on mental health, given economic disruptions, social isolation, and potential for trauma related to illness and death [1,2,3]. Although studies have documented psychological resilience in various populations during this period [4,5,6], others have found trends of worsening mental health in the general public [7,8,9,10,11,12,13,14].

Internationally, there were an estimated additional 76.2 million cases of anxiety disorders and 53.2 million cases of major depressive disorder in 2020 attributed to the COVID-19 pandemic, representing a 25.6% and a 27.6% increase in anxiety and major depressive disorder prevalence, respectively, compared to pre-pandemic prevalence [10]. The prevalence of posttraumatic stress disorder was also substantially higher in 2020, with 15.0–51.3% of individuals in community samples scoring above the threshold cutoff for probable posttraumatic stress disorder [13,14,15,16], compared to 3.5–4.7% in pre-pandemic years [17,18]. Among the United States (US) population, generalized anxiety, depression, and COVID-19-related posttraumatic stress disorder symptoms also increased in both prevalence and severity during 2020 [7,9,19]. Evidence from longitudinal studies indicates that some COVID-19-related mental health concerns have persisted beyond the onset of the pandemic [4,8,20]. For example, between 2020 and 2021, the prevalence of elevated depressive symptoms rose from 27.8% to 32.8% in the US [8].

Prior research has identified several demographic groups at increased risk for COVID-19-related mental health issues. Women [7,10,21,22,23,24,25], young adults [22,26,27,28,29,30], and the economically disadvantaged (i.e., low income or unemployed) [7,22,26,31] are three groups consistently found to be at a heightened risk for adverse mental health outcomes during the pandemic. Having children at home or having low familial or social support are other factors associated with higher levels of psychological distress [7,19,27,32,33]. COVID-19-related stressors associated with psychological stress include fear of infection [11,16,23]; suspected or confirmed infection [26,34]; knowing relatives, friends, and acquaintances who were infected, hospitalized, or died of COVID-19 [16,29,34]; financial uncertainty, economic loss, and impact on livelihood [11,12,35,36]; food insecurity [37,38]; loneliness [19,35,39,40]; quarantine or broader pandemic-related social isolation [23,26,28]; and essential worker status [41,42].

At the same time, there are important gaps in the understanding of COVID-19-related risk factors associated with mental health symptoms. Few prior studies have examined relationships between pandemic-related stress and suprathreshold risk of mental health symptoms. Studies utilizing clinical thresholds often focus on a single disorder or a limited set of demographic factors or COVID-19 stressors, limiting their ability to evaluate co-occurring influences and, consequently, to generate more comprehensive models. The objective of the present study was to assess the association of COVID-19-related stressors with elevated levels of anxiety, posttraumatic stress symptoms, and serious psychological distress in a US sample. We tested the hypothesis that individuals who reported COVID-19 related stressors would be more likely to report mental health symptoms above established cut-offs than those who did not report these exposures.

## 2. Materials and Methods

### 2.1. Survey Data Collection

The survey was distributed using the Amazon Mechanical Turk (MTurk) platform, as fully described in Monnig et al. (2021). Data collection focused on the five Northeast US states (Connecticut, Massachusetts, New Jersey, New York, Rhode Island) with the highest number of COVID-19 cases and deaths per capita at the time. The survey was released via MTurk to residents of these five states from 18 June to 19 July 2020. Data cleaning was performed to remove surveys that did not pass validity checks or were duplicate responses.

### 2.2. Participants

Eligibility criteria were as follows: 18 years of age or older; resident of CT, MA, NJ, NY, or RI; having an Amazon MTurk account (necessary for survey distribution). To obtain a diverse sample, quotas for age, gender, race, and ethnicity were instituted. Quotas were used because online surveys tend to recruit samples with overrepresentation of young, White, and/or female participants; and because we sought to oversample Black and Hispanic individuals due to evidence of disproportionate impacts on these groups of the pandemic [43,44]. Quotas were set to obtain a sample that was 40% non-Hispanic White, 25% Hispanic non-Black, 25% Black of any ethnicity; and 10% non-Hispanic non-White. Within each racial/ethnic group, age quotas were applied as follows: 10% 18–25 years old; 20% 25–35 years old; 20% 35–45 years old; 25% 45–55 years old; and 25% 55 years or older. Quotas were instituted for equal numbers by gender within each cell formed according to race/ethnicity and age. Participants were paid $10 for survey completion. The study was reviewed by Brown University Institutional Review Board and exempted from requiring approval due to minimal risk. Informed consent was obtained from all participants and the study was in accordance with the latest version of the Declaration of Helsinki.

### 2.3. Primary Exposures

COVID-19-related stressors were assessed as follows: (1) loss of employment (“Have you, or has anyone in your household experienced a loss of employment since March 13, 2020?”; no [reference]|yes); (2) knowing someone hospitalized (“Do you personally know anyone who has been hospitalized by the novel coronavirus, or COVID-19?”; no [reference]|yes); (3) essential worker status (“Are you an essential worker? An essential worker is someone whose work is critical to business operations and/or meeting basic human needs and who is required to attend work during the COVID-19 pandemic.”; no or not sure [reference]|yes); (4) worry about COVID-19 infection (“How worried are you about your risk of becoming infected with the novel coronavirus, or COVID-19?”; not worried or I have/had coronavirus or COVID-19 [reference]|somewhat worried|moderately worried|very worried); (5) children in the household under age 14 (“How many children currently live in your home at least part of the week who are under the age of 14?”; 0 [reference]|1 or more); (6) COVID-19 testing history (if endorsed testing for COVID-19 either by nasal swab or blood test, participant was asked, “What was your test result?”; not tested [reference]|positive|negative or unsure); (7) loneliness: total score on the 20 item UCLA Loneliness Scale (e.g., “I can find companionship when I want it”; “There are people who really understand me”; Response Range: 1 [never] to 4 [often]; *α* = 0.94) [45]; (8) barriers to environmental rewards (i.e., a lack of access to reinforcers and experience of unpleasant or aversive events): total score on the nine item Environmental Suppressor subscale of the Reward Probability Index (e.g., “Changes have happened in my life that have made it hard to find enjoyment”; “I have few financial resources, which limits what I can do”; Response Range: 1 [strongly disagree] to 4 [strongly agree]; *α* = 0.87) [46]; (9) food insecurity: total score on the nine item Household Food Insecurity Access Scale (e.g., “In the past four weeks, did you or any household member have to eat fewer meals in a day because there was not enough food?”; Response Range: either 0 [no] to 1 [yes] or 1 [rarely] to 3 [often]; *α* = 0.93) [47]. Knowing someone who died of COVID-19 was considered but not included in models due to high correlation with knowing someone hospitalized by COVID-19 (Pearson’s *r* = 0.616).

### 2.4. Primary Outcomes

Outcomes were standardized screening measures for mental health symptoms, as follows: (1) Generalized Anxiety Disorder-7 (GAD-7) for anxiety (e.g., “Over the past two weeks, how often have you been bothered by any of the following problems?: Feeling nervous, anxious or on edge”; N = 7; Response Range: 1 [not at all] to 4 [nearly every day]; α = 0.92) [48]; (2) Impact of Event Scale-Revised (IES-R) for posttraumatic stress symptoms (e.g., “I was aware that I still had a lot of feelings about it, but I didn’t deal with them”; “I felt watchful and on guard” N = 22; Response Range: 0 [not at all] to 4 [extremely]; α = 0.79–0.94) [49], wherein the COVID-19 pandemic was specified as the index event; (3) K6 scale for serious general psychological distress (e.g., “During the past 30 days, how often did you feel ... so depressed that nothing could cheer you up?”; N = 6; Response Range: 1 [all of the time] to 5 [none of the time]; α = 0.89) [50]. Scores were dichotomized to reflect absence/presence of risk for the relevant disorder (A Shapiro-Wilk test was conducted for each outcome variable to determine normality of distribution. Results indicated that all three variables violated the assumption of normality, *W* = 0.94, df = 1079, *p* < 0.001; *W* = 0.95, df = 1079, *p* < 0.001; *W* = 0.93, df = 1079, *p* < 0.001 for the GAD-7, K-6, and the IES-R, respectively. Histograms corroborated these findings and suggested that data for each outcome variable was positively skewed). Following previous research, thresholds for positive screenings were GAD-7 score ≥ 10. where the possible score range is 0–21 [48]; K6 score *≥* 13, where the possible score range is 0–24 [50]; and IES-R score *≥* 33, where the possible score range is 0–88 [51].

### 2.5. Sociodemographic Covariates

Sociodemographic covariates were age (continuous), income (eight ordered categories, treated as continuous), home ownership (no [reference]|yes), living alone (yes [reference]|no), education (high school or less [reference]|some college/2-year degree|college graduate|graduate or professional degree), race (White [reference]|Black or African American|Asian|multiracial or other race), ethnicity (Hispanic or Latino [reference]|not Hispanic or Latino), and gender (cisgender female [reference]|cisgender male|all other categories).

### 2.6. Statistical Analyses

Analyses tested the hypothesis that individuals who reported greater COVID-19-related stressors would be more likely to screen positive for risk of anxiety, posttraumatic stress symptoms, and serious psychological distress than those who did not report these stressors. The statistical analysis plan was preregistered following the collection of data but prior to data analysis at https://osf.io/zxsth (registered on 7 December 2021). Analyses were conducted in IBM SPSS Statistics, Version 27. We used logistic regression models to test the relationship between COVID-19 related stressors and anxiety, posttraumatic stress symptoms, and psychological distress.

Analyses were conducted in three stages. First, we fit three models (one for each outcome) with only sociodemographic covariates as the independent variables. Sociodemographic covariates that were significantly associated (*p*-value < 0.05) with the modeled outcome were included in the next step. Second, we fit nine partially adjusted logistic regression models, each of which included one COVID-19 exposure variable and the significant sociodemographic covariates from the prior stage. These partially adjusted models evaluated the significance of each individual exposure in the context of sociodemographic factors. Third, we fit three fully adjusted logistic regression models (one for each outcome) that included significant sociodemographic variables and all COVID-19 exposures simultaneously. Adjusted odds ratios (AORs) with bias-correlated and accelerated bootstrapped 95% confidence intervals (CI) are reported in the partially and fully adjusted models.

## 3. Results

### 3.1. Descriptive Statistics

In total, 1185 individuals completed the survey. The final analytic sample included 1079 participants after exclusions for invalid responding (*n* = 30), duplicate responses (*n* = 29), and missing covariates (*n* = 47).

Sample characteristics are shown in Table 1. The average sample was 40.9 years old and 49.7% female. Twenty-five percent of the sample identified as Hispanic. Sixty-nine percent of the sample identified as White, and 24.6% identified as Black or African American. Sample means ± standard deviations were 7.0 ± 5.6 on the GAD-7, 28.4 ± 22.2 on the IES-R, and 7.3 ± 5.5 on the K6. Percentages of the sample meeting risk thresholds were 34.8% on the GAD-7 for anxiety, 41.2% on the IES-R for posttraumatic stress reactions, and 15.8% on the K6 for serious psychological distress.

### 3.2. Correlations among Study Variables

Correlations among all continuous variables included in main analyses are reported in Table 2.

### 3.3. Step 1—Sociodemographic Covariates

Results of the models with sociodemographic covariates only are shown in Table 3. The sociodemographic characteristics statistically associated with anxiety were age, household income, living alone, education, race, ethnicity, and gender. Sociodemographic covariates statistically associated with COVID-19-related posttraumatic stress symptoms were age, income, living alone status, education, race, and ethnicity. Only income, home ownership, and race were significantly associated with serious psychological distress.

### 3.4. Step 2—Partially Adjusted Models with Significant Sociodemographic Covariates and Individual COVID-19-Related Stressors

Results of the partially adjusted models are shown in Table 4. The odds of anxiety were significantly higher in those with loss of employment; in those who knew someone hospitalized by COVID-19; in essential workers; in those moderately worried or very worried about risk of COVID-19 infection; in those with one or more children under 14 years old at home; in those with either a positive test result or a negative test result, relative to those not tested; in those with greater loneliness scores; and in those with greater barriers to environmental rewards and greater food insecurity. Similarly, all COVID-19-related stressors were associated with higher odds of posttraumatic stress symptoms related to COVID-19.

Odds of severe psychological distress were higher in those with loss of employment; in those very worried about risk of infection; in those with a positive test result, relative to those not tested; and in those with higher loneliness scores, greater barriers to environmental rewards, and greater food insecurity.

### 3.5. Step 3—Fully Adjusted Models with Significant Sociodemographic Covariates and All COVID-19-Related Stressors

Results of the fully adjusted models are shown in Table 5. The odds of anxiety were higher for essential workers; those very worried about risk of infection, compared to those who were not worried or those who had had COVID-19; and for those with greater loneliness scores, greater barriers to environmental rewards, and with greater food insecurity. Individuals of “All other gender categories” also had greater odds of anxiety than those who identified as cisgender female (AOR = 2.77; CI = 1.03, 10.14). Odds of posttraumatic stress symptoms were higher for essential workers; for those moderately or very worried about risk of COVID-19 infection; for those with greater loneliness, greater barriers to environmental rewards, and greater food insecurity. Importantly, the odds of posttraumatic stress in response to COVID-19 in those moderately worried or very worried about risk of COVID-19 infection were 3.51 times and 6.90 times greater, respectively, than the odds of posttraumatic stress in those who reported that they were not worried or already had COVID-19. Individuals not living alone (AOR = 2.19, CI = 1.10, 4.82) and those having a college degree (AOR = 1.92; CI = 1.05, 4.24) were significantly more likely to have posttraumatic stress symptoms, whereas those who identified as Asian (AOR = 0.41, CI = 0.16, 0.90), multiracial/other race (AOR = 0.19, CI = 0.08, 0.36), or non-Hispanic (AOR = 0.63, CI = 0.41, 0.92) had lower odds. Odds of serious psychological distress were higher for those with loss of employment, those with greater loneliness, and those with greater barriers to environmental rewards. No other stressors or covariates were associated with serious psychological distress.

## 4. Discussion

This study examined factors associated with anxiety, posttraumatic stress reactions, and general psychological distress in a diverse sample of participants several months into the COVID-19 pandemic. The geographical focus was five Northeast states with the highest rates of COVID-19 infection and deaths in the US at the time of the survey [52]. Our main finding was that, as hypothesized, experiencing COVID-19-related stressors was associated with a greater mental health burden. Specifically, when evaluated individually in the context of sociodemographic factors, all selected COVID-19-related stressors evaluated herein (i.e., loss of employment, knowing someone hospitalized by COVID-19, being an essential worker, worrying about infection, having one or more children under age 14 at home, COVID-19 testing, loneliness, barriers to environmental rewards, food insecurity) were associated with risk for one or more negative mental health outcomes.

When examined in models fully adjusted for sociodemographic covariates and all COVID-19-related stressors, specific factors stood out with respect to each mental health outcome. Anxiety, reported by 34.8% of our sample, was associated with essential worker status, worry about infection, loneliness, barriers to environmental rewards, and food insecurity. Posttraumatic stress symptoms related to the pandemic, found in 41.2% of our sample, were associated with essential worker status, worry about infection, loneliness, barriers to environmental rewards, and food insecurity. Finally, severe psychological distress, endorsed by 15.8% of our sample, was associated with loss of employment, loneliness, and barriers to environmental rewards. These findings are largely consistent with previous research demonstrating that COVID-19-related economic impact [11,12,35,36], food insecurity [37,38], essential worker status [41,42], worrying about infection [11,16,23], knowing someone hospitalized by COVID-19 [16,29,34], and loneliness [19,35,39,40] were risk factors for adverse mental health outcomes during the pandemic.

However, by evaluating numerous sociodemographic variables and COVID-19-related stressors concurrently across three symptomatic presentations, the present study also serves to advance understanding of these relations in two key ways. Our approach allowed us to identify COVID-19-related stressors that contributed independently to anxiety, COVID-19-related traumatic stress reactions, and severe psychological distress, accounting for other stressors and sociodemographic covariates. From this, overarching trends for each mental health outcome were identified. Our results suggest anxiety was a function of risk and exposure, as well as isolation and deprivation, while occupational exposure, isolation, and deprivation appeared to be the greatest risk factors for post-traumatic stress reactions to the COVID-19 pandemic. Finally, economic insecurity, in addition to isolation and lack of environmental rewards, appeared to be a major contributor to severe psychological distress.

We were also able to identify trends across mental health outcomes. Loneliness and barriers to environmental reward were risk factors across all three sets of mental health symptoms, suggesting that isolation and a lack of access to natural reinforcers (e.g., positive socialization, pleasant experiences) posed the most ubiquitous risk to mental health in our sample. These trends are in line with the broader literature, which has demonstrated social isolation and environmental deprivation to be deleterious to mental health across time [53,54] and to even have the potential to exacerbate the adverse effects of some forms of economic disadvantage (e.g., food insecurity) on mental health [55].

### Limitations

A key limitation is the approach to assessment of post-traumatic stress reactions related to COVID-19. In our survey, the COVID-19 pandemic was specified as the index event on the IES-R. However, the IES-R is not intended to be a diagnostic tool, and experiencing the COVID-19 pandemic does not necessarily constitute a Criterion A event (i.e., entailing threat of death or serious injury or assault), as required for diagnosis of PTSD. As discussed in recent publications [56,57,58,59], any survey-based approach that lacks assessment of a Criterion A event is likely to yield elevated rates of post-traumatic stress symptoms. Other potential downsides of a focus on post-traumatic stress symptoms are overlooking adjustment disorder as an alternative diagnosis and failing to account for human resilience to potentially traumatic situations.

While we attempted to recruit a sample that was diverse with regard to race, ethnicity, and age, and to balance gender, our sample is not representative of the population of the five Northeast US states that our study was based in. Consequently, findings may not be generalizable. As with any self-report data, inattentive or inaccurate responding is possible. However, our survey methodology did include checks for inattention and invalid response patterns. Due to the cross-sectional nature of the study, we cannot assume temporal precedence of stressors with regard to development of mental health symptoms. It is likely that relationships are bidirectional. For instance, it is possible that individuals with high levels of anxiety prior to the pandemic were predisposed to experience higher levels of anxiety about COVID-19 infection, and that the pandemic increased anxiety in some with higher baseline levels [60,61].

## 5. Conclusions

The findings add to understanding of the pandemic’s ramifications in a diverse sample located in the US states most severely affected by COVID-19, and at a point relatively early in the pandemic. The findings carry implications for identifying individuals in need of additional mental health and instrumental support. Those experiencing elevated levels of worry about COVID-19, loneliness, material deprivation, loss of employment, and barriers to environmental rewards were more likely to have higher risk for at least one of the conditions studied, namely, anxiety, posttraumatic stress, or severe psychological distress. Loneliness and barriers to environmental rewards were associated with increased odds of each mental health condition after accounting for all stressors and sociodemographic factors, suggesting that an inability to socialize and form positive new experiences posed the most global risk to mental health for our sample. These results can be used to inform the development of targeted screening and intervention tools to address the mental health burden of the COVID-19 pandemic. They also suggest that preparation efforts for future pandemics should give attention to devising novel and safe means for increasing interaction and pleasant life experiences among those required to practice social distancing, as doing so may serve to substantially mitigate the mental health burden post-pandemic.

## Figures and Tables

**Table 1:** Participant characteristics (N = 1079).

Variable	Value	Recode Information
Sociodemographics		
Age, mean (SD)	40.9 (13.5)	Not applicable
Sex assigned at birth, n (%)		Recoded to a combined sex and gender variable as follows: Cisgender male if sex = “male” and gender = “man” and no other gender endorsed|Cisgender female if sex = “female” and gender = “woman” and no other gender endorsed|Other for all other categories and combinations.
Male	536 (49.7)	
Female	536 (49.7)	
Prefer not to answer	7 (0.6)	
Gender (all that apply), n (%)		
Man	525 (48.7)	
Woman	543 (49.7)	
Transgender, Non-binary, Other, or Prefer not to answer ^†^	18 (1.7)	
Race (all that apply), n (%)		Recoded to 4 categories as follows: White if race = “White” and no other race endorsed|Black or African American if race = “Black or African American” and no other race endorsed|Asian if race = any of Asian Indian, Chinese, Filipino, Japanese, Korean, Vietnamese, or Other Asian and no other race endorsed|Multiracial or other for all other endorsed categories and combinations.
White	740 (68.6)	
Black or African American	265 (24.6)	
American Indian or Alaska Native	26 (2.4)	
Asian Indian	33 (3.1)	
Chinese	31 (2.9)	
Filipino	6 (0.6)	
Japanese	3 (0.3)	
Korean	7 (0.6)	
Vietnamese	1 (0.1)	
Other Asian	14 (1.3)	
Native Hawaiian	2 (0.2)	
Guamanian or Chamorro	1 (0.1)	
Other Pacific Islander	5 (0.5)	
Ethnicity (all that apply), n (%)		Ethnicity was dichotomized to Not Hispanic|Hispanic
Not of Hispanic, Latino/Latina, or Spanish origin	805 (74.6)	
Mexican, Mexican American, or Chicano/Chicana	75 (7.0)	
Puerto Rican	41 (3.8)	
Cuban	14 (1.3)	
Other Hispanic, Latino/Latina, or Spanish origin	155 (14.4)	
Education, n (%)		Not applicable
High school or less	106 (9.8)	
Some college/2-year degree	214 (19.8)	
College graduate/4-year degree	545 (50.5)	
Graduate or professional degree	214 (19.8)	
Annual household income, n (%)		Not applicable
<$25,000	121 (11.2)	
$25,000–$34,999	122 (11.3)	
$35,000–$49,999	150 (13.9)	
$50,000–$74,999	286 (26.5)	
$75,000–$99,999	188 (17.4)	
$100,000–$149,999	139 (12.9)	
$150,000–$199,999	39 (3.6)	
≥$200,000	34 (3.2)	
Household size, n (%)		Dichotomized to living alone|Not living alone
1 person	138 (12.8)	
2 people	265 (24.6)	
3 people	243 (22.5)	
4 people	288 (26.7)	
5 people	104 (9.6)	
6 or more	41 (3.8)	
Home ownership (yes), n (%)	505 (46.8)	Not applicable
COVID-19-related stressors		
Loss of employment since 13 March 2020 (yes), n (%)	447 (41.4)	Not applicable
Know someone hospitalized by COVID-19 (yes), n (%)	393 (36.4)	Not applicable
Essential worker, n (%)		Dichotomized to Not sure or No|Yes
Not sure	55 (5.1)	
No	637 (59.0)	
Yes	387 (35.9)	
Worry about infection, n (%)		Recoded to Not worried or already have/had COVID-19|Somewhat worried|Moderately worried|Very worried
Already have/had COVID-19	7 (0.6)	
Not worried	138 (12.8)	
Somewhat worried	304 (28.2)	
Moderately worried	360 (33.4)	
Very worried	270 (25.0)	
One or more children in the household under age 14 (yes), n (%)	398 (36.9)	Not applicable
COVID-19 testing history		Not applicable
Not tested	828 (76.7)	
Tested, negative result or unsure	207 (19.2)	
Tested, positive result	44 (4.1)	
UCLA Loneliness Scale, mean (SD)	22.4 (16.0)	Not applicable
Reward Probability Index, Environmental Suppressors subscale, mean (SD)	22.0 (5.8)	Not applicable
Household Food Insecurity Access Scale, mean (SD)	2.5 (4.6)	Not applicable

^†^ Note: These categories were asked separately but are combined here to prevent reporting of cell sizes < 5. Abbreviation: SD = standard deviation.

**Table 2:** Correlations among continuous variables included in main analyses.

	1	2	3	4	5	6	7	8	9
1. Age	---	0.02	−0.02	−0.13 **	−0.14 **	−0.13 **	−0.05	−0.15 **	−0.13 **
2. Household Income		---	−0.06 *	−0.24 **	−0.33 **	−0.18 **	−0.22 **	−0.19 **	−0.18 **
3. Worry About COVID-19 Infection			---	0.22 **	0.28 **	0.31 **	0.21 **	0.29 **	0.40 **
4. UCLA Loneliness Scale Score				---	0.69 **	0.62 **	0.37 **	0.60 **	0.62 **
5. Environmental Suppressors					---	0.61 **	0.39 **	0.60 **	0.63 **
6. GAD 7-Item Sum Score						---	0.39 **	0.68 **	0.72 **
7. Food Insecurity							---	0.36 **	0.45 **
8. K6 Sum Score								---	0.59 **
9. IES-R 22 Item Sum Score									---

Note: Continuous scores for GAD-7, K6, and IES-R were used when generating this correlation matrix, but dichotomous scores were used in main analyses. * *p* < 0.05; ** *p* < 0.01.

**Table 3:** Results of logistic regression models testing associations of sociodemographic characteristics with mental health symptoms.

	GAD-7	IES-R	K6
	AOR (95% CI)	AOR (95% CI)	AOR (95% CI)
**Age**	**0.99 (0.98, 0.99)**	**0.98 (0.97, 0.99)**	1.00 (0.98, 1.01)
**Household income**	**0.82 (0.75, 0.89)**	**0.77 (0.71, 0.83)**	**0.89 (0.80, 0.99)**
**Home ownership (Ref: No)**			
Yes	0.80 (0.60, 1.08)	1.17 (0.87, 1.61)	**0.59 (0.40, 0.85)**
**Living alone (Ref: Yes)**			
No	**2.32 (1.46, 3.67)**	**2.89 (1.82, 4.97)**	1.32 (0.77, 2.25)
**Education (Ref: High school or less)**			
Some college/2-year degree	0.78 (0.45, 1.33)	1.32 (0.75, 2.43)	1.18 (0.62, 2.25)
College graduate	1.53 (0.95, 2.46)	**2.47 (1.44, 4.61)**	0.91 (0.50, 1.65)
Graduate or professional degree	1.11 (0.64, 1.92)	**1.92 (1.09, 3.65)**	1.09 (0.55, 2.15)
**Race (Ref: White)**			
Black or African American	**1.70 (1.23, 2.33)**	**1.78 (1.27, 2.60)**	**2.09 (1.44, 3.03)**
Asian	0.64 (0.35, 1.17)	**0.44 (0.21, 0.78)**	0.76 (0.35, 1.65)
Multiracial or other race	0.72 (0.39, 1.35)	**0.39 (0.19, 0.72)**	0.37 (0.13, 1.08)
**Ethnicity (Ref: Hispanic or Latino)**			
Not Hispanic or Latino	**0.50 (0.37, 0.68)**	**0.33 (0.23, 0.45)**	0.98 (0.66, 1.46)
**Gender (Ref: Cisgender female)**			
Cisgender male	1.11 (0.84, 1.46)	1.08 (0.82, 1.45)	0.90 (0.63, 1.26)
All other categories	**2.93 (1.38, 6.20)**	1.66 (0.68, 4.23)	0.94 (0.36, 2.40)

Note: Categorical variable names and reference categories, where applicable, are bolded and are followed by subcategories within each main category. Bolded AORs indicate statistical significance, i.e., a bootstrapped 95% CI that does not include 1.00.

**Table 4:** Partially adjusted logistic regression models testing associations of sociodemographic characteristics and individual COVID-19-related stressors with mental health symptoms.

	GAD-7	IES-R	K6
	AOR (95% CI)	AOR (95% CI)	AOR (95% CI)
**Loss of employment** **(Ref: No)**			
Yes	**2.32 (1.74, 3.21)**	**2.44 (1.77, 3.43)**	**2.40 (1.67, 3.53)**
**Know someone hospitalized** **(Ref: No)**			
Yes	**1.95 (1.47, 2.66)**	**2.19 (1.65, 2.99)**	1.37 (0.98, 1.94)
**Essential worker status** **(Ref: No or not sure)**			
Yes	**2.20 (1.65, 3.01)**	**2.60 (1.93, 3.65)**	1.21 (0.86, 1.68)
**Worry about COVID-19 infection** **(Ref: Not worried or have/had coronavirus or COVID-19)**			
Somewhat worried	1.71 (0.98, 3.29)	**1.96 (1.08, 3.88)**	1.31 (0.64, 3.14)
Moderately worried	**2.36 (1.39, 4.47)**	**4.79 (2.80, 9.83)**	1.67 (0.86, 3.99)
Very worried	**4.89 (2.80, 9.63)**	**11.15 (6.04, 25.00)**	**3.24 (1.69, 7.85)**
**Children in the household under** **age 14 (Ref: 0)**			
1 or more	**1.95 (1.42, 2.72)**	**2.56 (1.89, 3.55)**	1.27 (0.91, 1.79)
**COVID-19 testing history** **(Ref: Not tested)**			
Positive	**2.80 (1.40, 6.53)**	**4.90 (1.88, 23.13)**	**2.24 (1.05, 4.28)**
Negative or unsure	**1.76 (1.25, 2.50)**	**2.49 (1.74, 3.74)**	1.37 (0.92, 2.03)
**Loneliness**	**1.08 (1.07, 1.10)**	**1.09 (1.08, 1.11)**	**1.06 (1.05, 1.08)**
**Barriers to environmental rewards**	**1.27 (1.22, 1.35)**	**1.29 (1.24, 1.36)**	**1.20 (1.15, 1.26)**
**Food insecurity**	**1.18 (1.14, 1.24)**	**1.25 (1.18, 1.35)**	**1.10 (1.06, 1.14)**

Note: Models are adjusted for different sociodemographic covariates due to different significant results in the preliminary logistic regression model testing associations of sociodemographic characteristics for each outcome. Covariates included in each model are as follows: (1) GAD-7: age, household income, living alone status, education, race, ethnicity, and gender; (2) IES-R: age, household income, living alone status, education, race, and ethnicity; (3) K6: household income, home ownership, and race. Categorical variable names and reference categories, where applicable, are bolded and are followed by subcategories within each main category. Bolded AORs indicate statistical significance, i.e., a bootstrapped 95% CI that does not include 1.00.

**Table 5:** Fully adjusted logistic regression models testing associations of sociodemographic characteristics and COVID-19-related stressors with mental health symptoms.

	GAD-7	IES-R	K6
	AOR (95% CI)	AOR (95% CI)	AOR (95% CI)
**Loss of employment (Ref: No)**			
Yes	1.36 (0.93, 2.05)	1.34 (0.86, 2.08)	**1.73 (1.11, 2.81)**
**Know someone hospitalized (Ref: No)**			
Yes	1.29 (0.89, 1.88)	1.30 (0.84, 2.05)	0.90 (0.57, 1.36)
**Essential worker status (Ref: No or not sure)**			
Yes	**1.55 (1.05, 2.31)**	**1.78 (1.15, 2.82)**	0.74 (0.47, 1.12)
**Worry about COVID-19 infection** **(Ref: Not worried or have/had coronavirus or COVID-19**			
Somewhat worried	1.27 (0.66, 2.74)	1.48 (0.66, 3.72)	1.09 (0.50, 2.65)
Moderately worried	1.40 (0.75, 2.92)	**3.51 (1.62, 8.99)**	1.19 (0.57, 2.83)
Very worried	**2.26 (1.16, 5.19)**	**6.90 (2.99, 20.70)**	1.86 (0.87, 4.78)
**Children in the household under age 14 (Ref: 0)**			
1 or more	1.12 (0.75, 1.65)	1.54 (0.995, 2.50)	0.72 (0.46, 1.14)
**COVID-19 testing history (Ref: Not tested)**			
Positive	0.52 (0.22, 1.26)	1.06 (0.36, 5.30)	0.87 (0.31, 2.15)
Negative or unsure	0.99 (0.63, 1.52)	1.31 (0.80, 2.17)	0.91 (0.52, 1.51)
**Loneliness**	**1.04 (1.03, 1.07)**	**1.06 (1.04, 1.09)**	**1.04 (1.02, 1.06)**
**Barriers to environmental rewards**	**1.15 (1.09, 1.22)**	**1.12 (1.06, 1.19)**	**1.10 (1.03, 1.18)**
**Food insecurity**	**1.07 (1.03, 1.14)**	**1.10 (1.04, 1.19)**	1.03 (0.99, 1.08)

Note: Models are adjusted for different sociodemographic covariates due to different significant results in the preliminary logistic regression model testing associations of sociodemographic characteristics for each outcome. Covariates included in each model are as follows: (1) GAD-7: age, household income, living alone status, education, race, ethnicity, and gender; (2) IES-R: age, household income, living alone status, education, race, and ethnicity; (3) K6: household income, home ownership, and race. Categorical variable names and reference categories, where applicable, are bolded and are followed by subcategories within each main category. Bolded AORs indicate statistical significance, i.e., a bootstrapped 95% CI that does not include 1.00.

## Data Availability

The raw data supporting the conclusions of this article will be made available by the authors, without undue reservation.
